# The interplay between natural and sexual selection in the evolution of sexual size dimorphism in *Sceloporus* lizards (Squamata: Phrynosomatidae)

**DOI:** 10.1002/ece3.2572

**Published:** 2017-01-13

**Authors:** Víctor H. Jiménez‐Arcos, Salomón Sanabria‐Urbán, Raúl Cueva del Castillo

**Affiliations:** ^1^ UBIPROLaboratorio de EcologíaUniversidad Nacional Autónoma de México, FES IztacalaMexico CityMexico

**Keywords:** dimorphism, fecundity, Lizards, natural selection, Rensch's rule, *Sceloporus*, sexual selection

## Abstract

Sexual size dimorphism (SSD) evolves because body size is usually related to reproductive success through different pathways in females and males. Female body size is strongly correlated with fecundity, while in males, body size is correlated with mating success. In many lizard species, males are larger than females, whereas in others, females are the larger sex, suggesting that selection on fecundity has been stronger than sexual selection on males. As placental development or egg retention requires more space within the abdominal cavity, it has been suggested that females of viviparous lizards have larger abdomens or body size than their oviparous relatives. Thus, it would be expected that females of viviparous species attain larger sizes than their oviparous relatives, generating more biased patterns of SSD. We test these predictions using lizards of the genus *Sceloporus*. After controlling for phylogenetic effects, our results confirm a strong relationship between female body size and fecundity, suggesting that selection for higher fecundity has had a main role in the evolution of female body size. However, oviparous and viviparous females exhibit similar sizes and allometric relationships. Even though there is a strong effect of body size on female fecundity, once phylogenetic effects are considered, we find that the slope of male on female body size is significantly larger than one, providing evidence of greater evolutionary divergence of male body size. These results suggest that the relative impact of sexual selection acting on males has been stronger than fecundity selection acting on females within *Sceloporus* lizards.

## 1 Introduction

In animal species that reproduce sexually, adult males and females often differ in body size. This difference is termed sexual size dimorphism (SSD) and generally evolves because body size is commonly related to reproductive success through different pathways in females and males (Blanckenhorn, 2005; Fairbairn, Blanckenhorn, & Székely, 2007). In females, body size is strongly correlated with fecundity, whereas in males, body size is correlated with mating success. As result of these differences, the body size that conveys maximal fitness often differs between the sexes (Fairbairn et al., 2007). The impact of sexual selection on SSD has been well established in many studies of individual species as well as in many phylogenetically controlled comparisons among species (Andersson, 1994; Fairbairn, 1997; Fairbairn et al., 2007). In addition, fecundity selection favors large female body size in species where females mature large numbers of eggs or live young within their abdomens, as in most fish, insects, and spiders (Blanckenhorn, 2005; Fairbairn, 1997; Fairbairn et al., 2007; Ruckstuhl & Neuhaus, 2005). SSD also can arise through ecological niche divergence, such as sex‐specific foraging/dispersal strategies or adaptations to reduce intersexual trophic competition (reviews in Blanckenhorn, 2005; Fairbairn, 1997; Fairbairn et al., 2007; Hedrick & Temeles, 1989; Reiss, 1989; Ruckstuhl & Neuhaus, 2005; Shine, 1989). However, it is unlikely that niche divergence between males and females is truly independent of sexual divergence in reproductive roles (Butler & Losos, 2002; Butler, Schoener, & Losos, 2000; Fairbairn et al., 2007).

In many vertebrate and invertebrate taxa, the magnitude of SSD changes systematically with mean body size, either increasing or decreasing as body size increases (Fairbairn et al., 2007; Webb & Freckleton, 2007). The former pattern is common in species where males are larger than females, while the latter occurs commonly in species in which females are the larger sex. Both patterns are explained by greater evolutionary divergence in male size, compared with female size; a pattern known as Rensch's rule (Fairbairn, 1997; Rensch, 1950). This allometric trend is usually attributed to sexual selection acting on male body size (Fairbairn et al., 2007; Stillwell et al., 2010). The converse trend, where female size varies more than male size, is less common, but seems to be the result of strong fecundity selection acting on females (Fairbairn et al., 2007; Foellmer & Moya‐Laraño, 2007; Webb & Freckleton, 2007). Lizards exhibit a broad range of SSD. However, in the majority of species, males are larger than females (Cox, Butler, & John‐Alder, 2007; Cox, Skelly, & John‐Alder, 2003), mainly because body size often determines success in agonistic encounters, and it is correlated with dominance and territoriality (Carpenter, 1995; McMann, 1993; Molina‐Borja, Padron‐Fumero, & Alfonso‐Martin, 1998; Perry et al., 2004). Nonetheless, in some species, females are larger than males, suggesting that fecundity selection may have favored the evolution of large female body size because it may allow females to (1) accommodate more offspring (Cox et al., 2003; Stuart‐Fox, 2009; Zamudio, 1998) and (2) increase the capacity for storing energy to be invested in reproduction (Calder, 1984; Pincheira‐Donoso & Tregenza, 2011).

Lizards species can be oviparous or viviparous (Blanckenhorn, 2000; Méndez–de la Cruz, Villagrán‐Santa Cruz, & Andrews, 1998). In some viviparous species, the embryos develop in a placenta with little or no shell forming, whereas in other species, the female retains the eggs within the uterus until development is complete. In any case, because placental gestation or extended egg retention requires more space within the abdominal cavity associated with an increased gestation period (Pincheira‐Donoso & Tregenza, 2011; Qualls & Shine, 1995), it has been suggested that the females of viviparous lizards possess larger body size or greater abdomens than their oviparous relatives (Braña, 1996; Scharf & Meiri, 2013; Yan‐Yan et al., 2012).

The lizard genus *Sceloporus* serves as an excellent example of SSD in lizards. This is a widely distributed genus (from southwestern Canada to northern Panama), which can be found in several environments and along broad altitudinal ranges (0 to >4,000 m; Sites et al., 1992; Smith, 1939). There are both oviparous and viviparous species in the genus (Méndez–de la Cruz, Villagrán‐Santa Cruz & Andrews, 1998). In the majority of species, males are the larger sex and exhibit a conspicuous coloration formed by belly and gular patches. However, these characteristics are also present in the females of some species within the group (Calisi & Hews, 2007; Carpenter, 1978; Fitch, 1978; Jiménez‐Cruz et al., 2005; Köhler & Heimes, 2002; Ramírez‐Bautista & Pavón, 2009; Ramírez‐Bautista et al., 2008; Ramírez‐Bautista, Stephenson, Lozano, et al., 2012; Weiss, 2006). In addition, conspicuous coloration is also present on the dorsum, including the head, tail, and limbs (e.g., *Sceloporus minor, S. aureolus, S. horridus:* Köhler & Heimes, 2002; Stephenson & Ramírez‐Bautista, 2012). The sexual coloration in males, principally the belly and gular patches, is related to species recognition, territory defense, agonistic interactions, and courtship (Carpenter, 1978; Martins, 1994; Sites et al., 1992; Wiens, Reeder, & Nieto Montes de Oca, 1999), which suggests that sexual selection has generated much of the divergence among males and females in *Sceloporus* lizards. However, in other species, females are larger than males (Fitch, 1978), suggesting that in these species, selection on female fecundity has been stronger than sexual selection on males.

In this study, we explore the relationship between female body size, fecundity and reproductive modes, and the potential impact of these relationships on body size divergence between females and males of *Sceloporus* lizards. In addition, we tested Rensch's rule in order to evaluate the relative impact of sexual selection on the evolution of SSD, and we performed an ancestral character reconstruction to infer the evolutionary trends of SSD in these lizards. We expected differences in body size between oviparous and viviparous females and that these differences affect the body size relationships between the sexes. Nonetheless, if sexual selection has been the main force driving the evolution of SSD in *Sceloporus*, we predict that the regression of male size on female size will have a slope steeper than 1, following the Rensch's rule.

## 2 Methods

### 2.1 Data collection

Our study comprised data collected for 56 *Sceloporus* species, four *Urosaurus* species and *Petrosaurus thalassinus* for a total of 61 evolutionary units (*Urosaurus* and *P. thalassinus* were used as outgroup taxa). The *Sceloporus* species sampled included all major species groups of the genus (Leaché, 2010; Wiens et al., 2010); 41 species were oviparous and 20 were viviparous (Table 1). We performed a literature search for data on snout‐vent length (SVL; a standard measure used as a proxy for lizard size; Cox et al., 2003; Losos, 1990) for both females and males and clutch/litter sizes (number of eggs or embryos) for the species studied. We collected information from the literature by executing searches on Google Scholar using the terms “snout‐vent length,” “clutch size,” “litter size,” “number of eggs/embryos,” “sexual size dimorphism,” or “reproductive cycle” for a list of species of the genus *Sceloporus*, reported by Wiens, Kozak, and Silva (2013). Google Scholar was used as the search engine instead of other engines because it cataloged full‐text versions of published papers. Moreover, terms that were included in our search like “clutch size,” “litter size,” and “snout‐vent length” were not the principal focus of the papers, and the phrases were usually referred to only briefly. Thus, we were less likely to locate the pertinent information using literature databases that contain only keywords, titles, and abstracts (see Dornhaus, Powell, & Bengston, 2012). We excluded data in which the number of vitellogenics follicles were reported as part of clutch size, because the follicular atresia may occur in any stage of the ovogenesis, including previtellogenic and vitellogenic follicles, and thus does not represent an accurate estimation of clutch/litter size (Méndez‐de la Cruz et al., 2013). For species with data on more than one clutch per reproductive season, we used the average of all clutches reported in the literature.

**Table 1:** Mean snout‐vent length (SVL), clutch/litter size, and reproductive mode (O = oviparous and V = viviparous) for 56 *Sceloporus* species and five outgroup taxa

Species	SVL females (mm)		SVL males (mm)		Clutch size		Reproductive mode	References
*Petrosaurus thalassinus*	99.15 (71–110)	(44)	107.23 (80–152)	(44)	8.6 (4–18)	(10)	O	Goldberg and Beaman (2004)
*Sceloporus adleri*	63.11 (54–78.8)	(23)	65.28 (59–72)	(14)	6.57 (2–11)	(14)	V	Fitch (1978), Santos‐Bibiano (unpublished data)
*S. aeneus*	51.88 (43.4–59.1)	(194)	52.98 (43.4–62.8)	(138)	7.3 (7–12)	(32)	O	Jiménez‐Arcos (2013)
*S. angustus*	62.8 (61–66)	(5)	78.2 (65–86)	(6)	5.5 (4–7)	(5)	O	Goldberg (2014)
*S. arenicolus*	53.8 (49–62.2)	(339)	54.5 (49–64.9)	(507)	5 (4–6)	(?)	O	Fitzgerald et al. (2011)
*S. bicanthalis*	51.84 (42.4–58)	(85)	43.6 (42–53.2)	(42)	7.18 (3–9)	(68)	V	Rodríguez‐Romero et al. (2010), This study^a^
*S. chrysostictus*	51.3	(82)	53.95	(82)	2.4 (1–4)	(16)	O	Fitch (1985), Köhler and Heimes (2002)
*S. clarkii*	88.08 (72–120)	(57)	103 (91–138)	(56)	10.85 (1–24)	(39)	O	Fitch (1978, 1985), Parker and Pianka (1973)
*S. consobrinus*	68.4 (54–77)	(58)	60.5 (50–68)	(44)	9.9	(39)	O	Vinegar (1975a)
*S. couchii*	50	(36)	58	(32)	4	(?)	O	García de la Peña et al. (2004), Lemos‐Espinal and Smith (2007)
*S. cozumelae*	45.48 (41–57)	(33)	50.72 (43–60)	(57)	1.8	(12)	O	Fitch (1978)
*S. cryptus*	67.06 (58.5–76.6)	(8)	61.6 (58.9–68.5)	(6)	9 (6–12)	(4)	V	This study^b^
*S. cyanogenys*	63	(15)	66	(15)	16.45 (6–18)	(36)	V	Fitch (1985), García‐de la Peña, Castañeda, and Lazcano (2005)
*S. dugesii*	61.5 (50–78)	(91)	65.9 (50–98)	(73)	4.4 (1–10)	(27)	V	Ramírez‐Bautista and Dávila‐Ulloa (2009)
*S. edwardtaylori*	107	(?)	107	?	8.5 (8–9)	(2)	O	Köhler and Heimes (2002)
*S. for. formosus*	67.46 (50–83.3)	(113)	67.98 (50–87.4)	(99)	8.63 (6–18)	(16)	V	Ramírez‐Bautista and Pavón (2009), This study^b^
*S. for. scitulus*	66.49 (62.5–84.9)	(82)	70.88 (63.3–87.3)	(73)	6.04 (2–12)	(27)	V	Ramírez‐Pinilla et al. (2009), This study^a^
*S. gadoviae*	54.95 (45.7–57.2)	(6)	64.9 (69.6–73.5)	(6)	3.6 (1–5)	(20)	O	Lemos‐Espinal, Smith, and Ballinger (1999), This study^a^
*S. graciosus*	57.59 (48–69)	(197)	55.18 (48–63)	(182)	4.55 (1–10)	(381)	O	Burkholder and Tanner (1974), Fitch (1978, 1985), Tinkle (1973)
*S. grammicus*	56.05 (42.1–72.5)	(278)	60.06 (45–79.9)	(412)	5.35 (2–12)	(167)	V	Ramírez‐Bautista, Stephenson, Hernández‐Íbarra, et al. (2012), Ramírez‐Bautista, Stephenson, Lozano, et al. (2012), This study^a^
*S. grandaevus*	58.5 (58–59)	(2)	72.1 (67–78)	(5)	6.5 (6–7)	(2)	O	Goldberg (2014)
*S. horridus*	82.17 (60–100)	(46)	85.49 (52–118)	(82)	14 (7–18)	(16)	O	Valdéz‐González and Ramírez‐Bautista (2002), This study^a^
*S. hunsakeri*	64.13	(19)	73.96	(20)	7.5 (5–10)	(2)	O	Galina Tessaro et al. (2015)
*S. jalapae*	46 (42–50)	(24)	49.3 (45–62)	(17)	5.6 (4–8)	(10)	O	Ramirez‐Bautista et al. (2005)
*S. jarrovii*	66.21 (60–86)	(787)	69.67 (46–98)	(668)	7.35 (2–16)	(405)	V	Ballinger (1973), Gadsden and Estrada‐Rodríguez (2007)
*S. licki*	63.83	(13)	71.46	(24)	6	(?)	O	Galina Tessaro et al. (2015)
*S. macdougalli*	83.84 (72.5–95.4)	(29)	88.82 (81.8–92.5)	(7)	3.88 (2–5)	(9)	V	Martínez Bernal (2004)
*S. magister*	93.64 (80–120)	(54)	111.45 (80–140)	(53)	6.98 (2–12)	(43)	O	Fitch (1978, 1985)
*S. malachiticus*	75.49 (64–86)	(208)	79.12 (67–90)	(146)	6 (3–10)	(44)	V	Fitch (1978, 1985)
*S. megalepidurus*	44.99 (37–48)	(36)	47.28 (39–55)	(76)	2.04 (1–4)	(25)	V	Fitch (1978), Godinez‐Cano (1985)
*S. melanorhinus*	87.9 (62–98)	(30)	84.6 (62–95)	(32)	7.7 (5–9)	(12)	O	Ramirez‐Bautista et al. (2006)
*S. merriami*	48.13 (39–55)	(164)	52.24 (42–61)	(355)	4.33 (2–7)	(127)	O	Fitch (1978), Grant and Dunham (1990)
*S. minor*	65.65 (41.6–92.9)	(182)	70.32 (53.6–99.4)	(169)	6.09 (2–13)	(46)	V	Ramírez‐Bautista et al. (2008, 2014)
*S. mucronatus*	78.89 (56.5–102)	(170)	87.02 (55.2–111.2)	(146)	5.8 (2–13)	(49)	V	Ortega‐León et al. (2007), Villagrán‐Santa Cruz et al. (2009), This study^a^
*S. nelsoni*	52.14 (48–58)	(21)	60.15 (53–65)	(26)	6.25 (4–8)	(4)	O	Fitch (1978)
*S. occidentalis*	74.63 (68–87)	(43)	68.35 (61–81)	(46)	8.12 (3–14)	(243)	O	Fitch (1978), Herrel, Meyers, and Vanhooydonck (2002)
*S. ochoterenae*	44.39 (31–67)	(110)	48.23 (44–56)	(143)	6.77 (3–7)	(35)	O	Bustos‐Zagal et al. (2011), Smith and Lemos‐Espinal (2003)
*S. olivaceus*	93 (63–107)	(107)	82.9 (60–93)	(34)	14.3 (8–30)	(14)	O	Blair (1960)
*S. omiltemanus*	83.08	(39)	98.11	(25)	6.23 (6–8)	(13)	V	Ramírez‐Pinilla et al. (2009)
*S. orcutti*	92 (85–106)	(77)	102 (90–115)	(17)	11 (8–15)	(4)	O	Mayhew (1963)
*S. parvus*	46.85 (44.7–49)	(?)	50	(?)	3.8	(>2)	O	García‐Vázquez, Trujano‐Ortega, and Contreras‐Arquieta (2014), Lemos‐Espinal and Dixon (2013)
*S. pictus*	47.86 (44–52)	(7)	48.88 (47–51)	(8)	3.6 (2–6)	(5)	V	Fitch (1978)
*S. poinsettii*	89.45 (79–116)	(55)	96.79 (77–130)	(79)	10.5 (4–23)	(90)	V	Fitch (1978, 1985), Gadsden et al. (2005)
*S. pyrocephalus*	53.41 (47–62)	(88)	62.01 (50–75)	(84)	5.65 (4–9)	(24)	O	Fitch (1978), Ramírez‐Bautista and Olvera Becerril (2004)
*S. spi. caeruleopunctatus*	87.22 (77–96)	(18)	88.29 (82–99)	(17)	12.82 (8–19)	(23)	O	Calderón‐Espinosa, Andrews, and Méndez de la Cruz (2006), Fitch (1978)
*Sceloporus spi. spinosus*	91.11 (65.7–110.5)	(164)	92.66 (60–112)	(164)	14.09 (6–22)	(38)	O	Méndez de la Cruz et al. (2013), Ramírez‐Bautista, Stephenson, Hernández‐Íbarra, et al. (2012), Ramírez‐Bautista, Stephenson, Lozano, et al. (2012), Ramírez‐Bautista et al. (2014), Valdéz‐González and Ramírez‐Bautista (2002)
*S. scalaris*	51.25 (40–60)	(203)	45.53 (40–55)	(45)	8.28 (4–15)	(109)	O	Carbajal‐Márquez and Quintero‐Díaz (2013), Fitch (1978, 1985), Vitt (1977)
*S. siniferus*	49.88 (40–61)	(139)	52.49 (53–61)	(235)	4.94 (2–8)	(15)	O	Fitch (1978), Ramírez‐Bautista et al. (2015)
*S. smaragdinus*	62.24 (55–77)	(17)	67.22 (60–80)	(14)	4.2 (3–6)	(10)	V	Fitch (1978)
*S. subpictus*	66.47 (63.1–69)	(41)	63.54	(1)	13 (12–14)	(2)	V	This study^b^
*S. torquatus*	94.03 (65–110)	(4)	101.51 (43.2–115.9)	(37)	7.78 (3–17)	(84)	V	Feria Ortiz, Salgado Ugarte, and Nieto‐Montes de Oca (2001), Guillette and Méndez‐de la Cruz (1993), This study^a^
*S. tristichus*	63.3 (48–67)	(57)	55.9 (53–73)	(54)	7.2	(29)	O	Vinegar (1975b)
*S. undulatus*	61.11 (53–72)	(118)	55.78 (45–65)	(177)	8.02 (3–15)	(376)	O	Fitch (1978, 1985), Herrel et al. (2002)
*S. utiformis*	63.41 (51–73)	(104)	61.25 (45–84)	(122)	6.94 (3–10)	(31)	O	Fitch (1978), Ramírez‐Bautista and Gutiérrez‐Mayén (2003)
*S. variabilis*	52.65 (44–68)	(424)	61.99 (42–74)	(457)	3.92 (1–7)	(216)	O	Benabid (1994), Cruz‐Elizalde & Ramírez‐Bautista (2016 and references in table 6), Fitch (1978, 1985)
*S. virgatus*	63.81 (51–74.2)	(54)	50.42 (48–58)	(22)	9.44 (4–16)	(228)	O	Abell (1999), Herrel et al. (2002), Vinegar (1975a)
*S. woodi*	57.24	(64)	51.89	(78)	4.62 (2–8)	(231)	O	Jackson and Telford (1974), Williams (2010)
*Urosaurus bicarinatus*	45.84 (40–53)	(249)	49.66 (38–61)	(322)	6.26 (2–11)	(50)	O	Ramírez‐Bautista, Uribe‐Peña, and Guillette (1995), Ramirez‐Bautista and Vitt (1998)
*U. graciosus*	38.69 (44–66)	(60)	62.35 (42–66)	(42)	4.05 (2–10)	(25)	O	Fitch (1985), Vitt, Van Loben Sels, and Ohmart (1978)
*U. nigricaudus*	51.82 (44–60)	(121)	62.47 (57.2–65.4)	(42)	4.05 (2–6)	(25)	O	Romero‐Schmidt, Ortega‐Rubio, and Acevedo‐Beltran (1999)
*U. ornatus*	49.98 (45–58)	(14)	50.87 (47–60)	(34)	7.25 (2–12)	(1454)	O	Fitch (1985), Martin (1973), Van Loben Sels and Vitt (1984)

Size and clutch/litter size ranges are shown in parentheses below mean values. Numbers between parentheses refer to sample sizes. The symbol (?) represents a lack of sample size data in the literature.

a Only SVL data obtained in this study.

b Both SVL and litter size data obtained in this study.

In addition to this data set, we incorporated unpublished measurements collected by us from the individuals of ten species. Both SVL and litter size data were incorporated for *S. cryptus, S. formosus formosus,* and *S. subpictus* (all viviparous species). SVL data from both sexes were collected for *S. bicanthalis, S. formosus scitulus, S. gadoviae, S. grammicus, S. horridus, S. mucronatus,* and *S. torquatus*. Litter size was obtained from direct observations of females giving birth in captivity (see Bastiaans et al., 2014 for care details). Digital calipers were used to take SVL measurements to the nearest 0.1 mm (Mitutoyo CD‐15DC; Mitutoyo Corp., Tokyo, Japan). All lizards captured for this study were unharmed and released at their original capture locations following data collection.

The number of eggs or embryos was used as an estimation of fecundity. Prior to further analyses, all measurements were log_10_‐transformed to improve linear fits. In addition, we estimated a sexual size dimorphism index (SDI) on SVL following the Lovich and Gibbons (1992) criteria. This index expresses SSD as [(length of larger sex/length of smaller sex) − 1]. For convention, the SDI is arbitrarily changed to negative when males are the larger sex and positive when females are the larger sex (Cox et al., 2007).

### 2.2 Phylogenetic reconstruction

We inferred the phylogenetic relationships between the 56 studied species of *Sceloporus* using the nucleotide sequences of eight nuclear (BDNF, ECEL, PNN, PRLR, PTPN, R35, RAG1, TRAF6) and five mitochondrial genes (12S, 16S, ND1, ND2, ND4) available on GenBank. We also retrieved the same genetic information from five outgroup taxa which included four *Urosaurus* species*,* representing the sister group of *Sceloporus* (Leaché, 2010; Wiens et al., 2010) and *Petrosaurus thalassinus*. The number of species sampled for each gene was BDNF = 48, ECEL = 25, PNN = 47, PRLR = 27, PTPN = 26, R35 = 48, TRAF6 = 46, 12S = 57, 16S = 56, ND1 = 54, ND2 = 35, and ND4 = 57. All matrices were similar to previous studies (Leaché, 2010; Wiens et al., 2010). However, we treated the two subspecies of *S. formosus* (i.e., *S*. *formosus formosus* and *S*. *formosus scitulus*) as putative species based on previous evidence for distinct lineages (Pérez‐Ramos & Saldaña de La Riva, 2008; Wiens & Reeder, 1997). A similar situation is present in *S. spinosus* (with *S. spinosus spinosus* and *S. spinosus caeruleopunctatus*). Wiens et al. (2010) recognized these taxa as putative species, which was also supported by more recent evidence (Grummer et al., 2015). Our inclusion of these taxa as distinct evolutionary lineages was not an endorsement of their recognition as different species, but we did not want to ignore important previous taxonomic work on these groups (see Pérez‐Ramos & Saldaña de La Riva, 2008; Wiens & Reeder, 1997; Wiens et al., 2013).

We used MUSCLE algorithm (Edgar, 2004) to align each gene data set using the default parameters in the software MEGA (version 7; Kumar, Stecher, & Tamura, 2016). We then used the software MESQUITE (Maddison & Maddison, 2015) to combine the sequences of each gene, and to make the final concatenated matrix for all genes (see below). We provide the GenBank accession numbers of the sequences used in Appendix S1. Our concatenated alignment consisted of genetic information from 61 terminals (56 *Sceloporus* species, five outgroups taxa) and 11,113 characters. We estimated the best partition scheme and nucleotide substitution models for the data using the greedy algorithm of PARTITIONFINDER (version 1.1.1; Lanfear et al., 2014). We conducted a concatenated Bayesian inference (BI) analysis in MRBAYES (version 3.2.6; Ronquist et al., 2012) by applying the specific substitution model estimated for each partition. The BI analysis consisted of four independent runs, each with 10,000,000 generations and four chains, sampling every 1,000 generations. We used default priors for other parameters in the analysis. We assessed parameter convergence and proper mixing of independent runs using TRACER (version 1.6; Rambaut & Drummond, 2013). All parameter values sampled during the MCMC of the analysis resulted in ESS values greater than 200. We discarded 25% of the samples obtained prior to stability as burn‐in to obtain a final consensus tree (See Appendix S1 for details).

Our analysis only considered the phylogeny that resulted from a concatenated matrix of both mitochondrial and nuclear loci, utilizing a total evidence approach for *Sceloporus* species and outgroup taxa. Although this approach may be controversial because nuclear and mitochondrial genes may have incongruent histories due to incomplete lineage sorting and exhibit different substitutions rates (see Maddison, 1997), concatenated matrices have improved the resolution of the phylogenetic relationships of phrynosomatid lizards (Wiens et al., 2010). Moreover, our phylogenetic results were largely congruent with a recent phylogenetic study on *Sceloporus* that involved a wider taxonomic and genetic sampling, as well as different methodological approaches (concatenation and coalescent‐based methods) to infer phylogenetic relationships (Leaché et al., 2016).

### 2.3 Comparative analyses

We converted the molecular branch lengths from the Bayesian analysis to units of time using a penalized likelihood method (Sanderson, 2002). For branch length conversion, we used the R (version 3.1.3; R Core Team 2015) package “ape” (Paradis, Claude, & Strimmer, 2004) and performed all the comparative analysis on the resulting ultrametric phylogeny. For more details, see Appendix S1.

### 2.4 Reproductive modes, female body size, fecundity, and SDI

We used the phylogenetic generalized least squares (PGLS) model to test for an association between fecundity, body size, and reproductive mode. The PGLS approach incorporates phylogenetic information into linear models to account for the statistical nonindependence of residuals using a variance–covariance matrix (see Martins & Hansen, 1997) specified by the phylogeny. For all models, the maximum likelihood value of the weighting parameter λ was estimated simultaneously with the models (Gonzalez‐Voyer & Kolm, 2010; Revell, 2010). The λ parameter indicates whether trait evolution is independent of the phylogeny (λ = 0) or evolving according to Brownian motion (λ = 1). Intermediate values of λ suggest a process in which the effect of the given phylogeny is weaker than expected by Brownian motion evolution (Pagel, 1999). The models were fitted as implemented in the R package “caper” (Orme et al., 2012). The first model included fecundity (dependent variable), log_10_ SVL female (independent variable), and reproductive mode (categorical independent variable) as well as the interaction between SVL and reproductive mode. In order to evaluate the impact of fecundity on SDI, we first saved the residuals of the previous model and then constructed a model with SDI as the dependent variable, reproductive mode as a categorical independent variable, and the fecundity residuals as a covariate. We used the residuals to eliminate potential confounding effects associated with female body size.

### 2.5 Rensch's rule and ancestral reconstruction of SDI

Rensch's rule predicts that the slope of a regression of male body size on female body size will be steeper than 1. To test this prediction in the studied species, we used the phylogenetic independent contrasts method (PIC method; Felsenstein, 1985), as implemented by the R package “caper” (Orme et al., 2012) to control for the phylogenetic nonindependence of species (Harvey & Pagel, 1991). We examined the studentized residuals for outliers > |±3|, but found none in our data set. Also, in order to verify whether the standardized contrasts are independent from their estimated nodal values (see Felsenstein, 1985), we plotted the standardized contrasts against their estimated nodal values using the “plot” function provided by “caper”. Ultimately, we tested the allometric relationship between independent contrasts of log_10_ SVL male (dependent variable) and log_10_ SVL female (independent variable) by fitting major axis regression using the R package “smatr” (Warton et al., 2012). Major axis regression offers an accurate approach to test the null hypothesis of isometry (h_0_: β = 1), because both variables were measured on the same scale and residual variance is minimized in both *x* and *y* dimensions, rather than the *y* dimension only (Cox et al., 2007; Pincheira‐Donoso & Tregenza, 2011; Warton et al., 2006). Given that the mean value of contrasts is expected to be zero (Sanabria‐Urbán et al., 2015), we forced the major axis regression through the origin. We used the Wald statistic (*r*_*w*_) and confidence intervals (95%) of the slope to test the null hypothesis (see Warton et al., 2006). In addition, in order to explore the evolutionary trends in body size and SDI, we performed an ancestral character reconstruction following Revell (2013). This method estimates the maximum likelihood value for internal nodes and then interpolates the states along the branches of the tree (see Revell, 2013, 2014 for details). For the reconstruction and visualization of ancestral state reconstruction of SDI (see Figure 3), we used the R package “phytools” (Revell, 2012).

## 3 Results

### 3.1 Reproductive modes, body size, and fecundity

**Figure 1:**
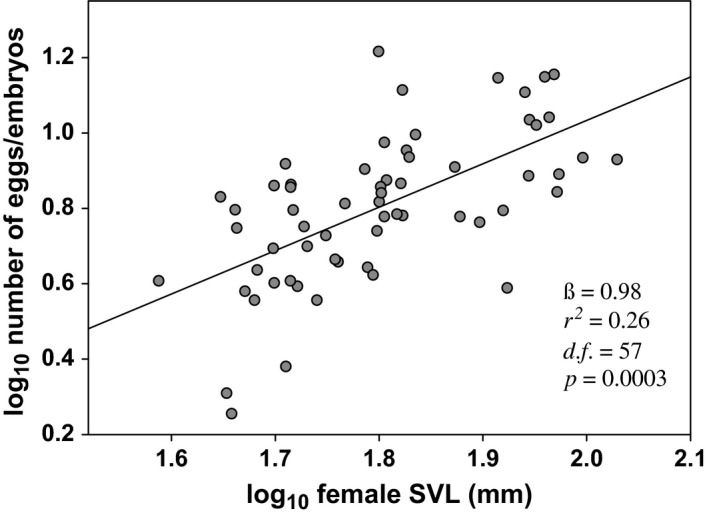
The relationship between the SVL of females and fecundity. Note this graph is shown only for illustrative purposes and was created with ordinary least squares linear model

After controlling for phylogenetic nonindependence among of the species studied, the results of the PGLS analysis were highly significant (*r* ^2^ = 0.3, *F*_3,57_ = 8.025, *p = *.0001). We found a strong and significantly positive relationship between body size and fecundity (β = 0.98 ± 0.26, *t *=* *3.801, *p *=* *.0003; Figure 1). Nonetheless, there were no differences in fecundity between reproductive modes (β = −0.15 ± 0.85, *t *=* *−0.174, *p *=* *.86). The interaction between reproductive modes and body size was not significant (β = 0.04 ± 0.47, *t *=* *0.086, *p *=* *.93), indicating a similar fecundity response to an increase in the body size of both oviparous and viviparous species. The model showed intermediate λ values (λ = 0.54), indicating a relatively weak phylogenetic effect on the relationships between body size and fecundity.

### 3.2 Reproductive modes, fecundity, and SDI

The results of PGLS analysis were not significant (*r* ^2^ = .003, *F*_3,57_ = 0.071, *p *=* *.98). There were no significant differences in the SDI of oviparous and viviparous lizards (β = 0.01 ± 0.05, *t *=* *0.433, *p *=* *.67). Similarly, there were no significant effects of fecundity residuals on SDI (β = −0.005 ± 0.08, *t *=* *−0.063, *p *=* *.95). The model showed a high λ value (λ = 0.95), indicating a strong phylogenetic effect on the relationships between fecundity residuals and SSD.

### 3.3 Rensch's rule and ancestral reconstruction of SDI

**Figure 2:**
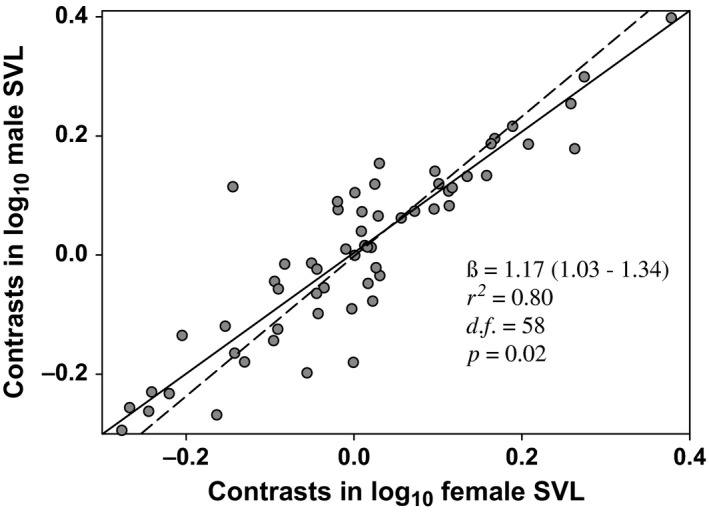
Independent contrasts of SVL of males as a function of SVL of females. The solid line indicates isometry (β = 1), while the dashed line denotes the allometric relationship between both variables as fitted by major axis regression. Values in parentheses indicate the upper and lower confidence interval (95%) for the slope and *p* value the probability for a β > 1

**Figure 3:**
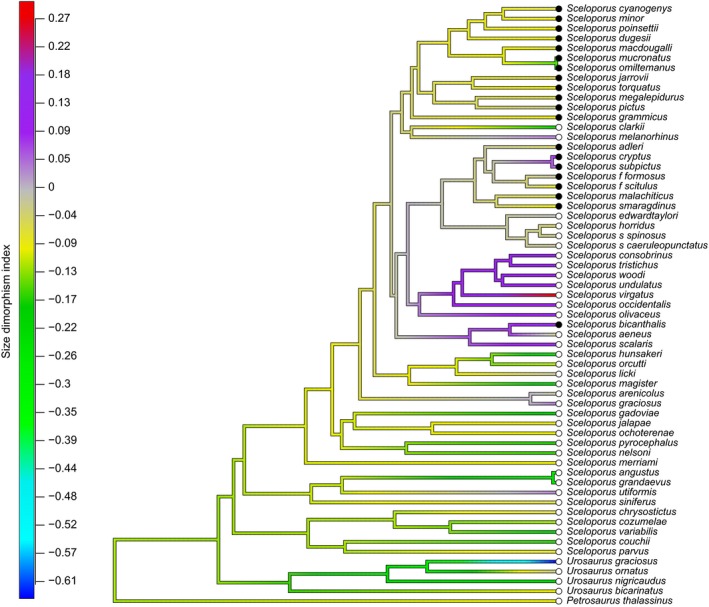
Maximum likelihood ancestral reconstruction of SDI for 56 species of *Sceloporus* and five outgroup taxa performed in R package “phytools” (Revell, 2012). For the analysis, we used the ultrametric phylogeny and the values of SDI estimated for each species. The values in the color ramp represent the range of SDI registered for the study species. Negative values indicate male‐biased SSD (blue to paleyellow) and positive values female‐biased SSD (palepurple to red). Open and filled circles indicate, respectively, oviparous and viviparous lizard species

The results of the major axis regression of independent contrasts indicated strong coevolution between females and males (*r *=* *.80; *df* = 58, *p *=* *.0001, Figure 2). The regression showed a slope significantly steeper than 1.0 (β = 1.17, *r*_*w*_ = .29, *p *=* *.02; Figure 2). Most of the taxa (46 species, 75%) showed male‐biased SSD, and 14 species (23%) showed some degree of female‐biased SSD. The males and females of only one species showed similar body sizes (*S. edwardtaylori*). The SDI reconstruction showed six independent origins of the female‐biased SSD. In a clade with male‐biased SSD (*formosus* group), the branch of *S. cryptus* and *S. subpictus* showed a female‐biased SSD. Other independent origin of female‐biased SSD was found in the *scalaris* (*S. bicanthalis* and *S. scalaris*) group. Another origin for *undulatus* group (*S. olivaceus*,* S. occidentalis*,* S. virgatus*,* S. woodi*,* S. undulatus*,* S. consobrinus,* and *S. tristichus*). Finally, three additional species independently evolved female‐biased SSD: *S. utiformis* (*utiformis* group), *S. graciosus* (*gracious* group), and *S*. *melanorhinus* (*clarkii* group; Figure 3).

## 4 Discussion

Once we control for phylogenetic effects, our results confirm a strong relationship between female body size and fecundity, suggesting that in *Sceloporus* lizards selection on fecundity has had a main role on the evolution of female body size. However, regardless of the reproductive mode (oviparous or viviparous), the size of females of *Sceloporus* is similar and has evolved in a similar fashion. We must point out that the similar response in the relationship of body size with increase in the clutch/litter size between both reproductive modes does not imply that the overall reproductive output (i.e., reproductive fitness of the female's life) is similar. The potential impact of fecundity selection on the different reproductive modes may be underestimated (Niewiarowski et al., 2004; Pincheira‐Donoso & Hunt, 2015; Shine, 2005). Oviparous species like *S. gadoviae*,* S. siniferus*,* S. undulatus,* and *S. variabilis* may have multiple clutches in a reproductive season (i.e., per year; Cruz‐Elizalde & Ramírez‐Bautista, 2016; Ramirez‐Bautista et al., 2005; Ramírez‐Bautista et al., 2015; Vinegar, 1975b), whereas other species like *S. magister*,* S. melanorhinus,* and *S. spinosus* have just one clutch per year, but they may have more than one reproductive event in their life (Méndez‐de la Cruz et al., 2013; Parker & Pianka, 1973; Ramirez‐Bautista et al., 2006; Valdéz‐González & Ramírez‐Bautista, 2002). On the other hand, all viviparous species have one litter per year, but in the majority of species, females can have several reproductive events (Méndez–de la Cruz et al., 1998; Ramírez‐Bautista et al., 2014; Villagrán‐Santa Cruz, Hernández‐Gallegos, & Méndez‐de La Cruz, 2009).

The differences in the gestation period between reproductive modes do not have any impact on the evolution of SSD, but according to the Renchs' rule, the slope of the regression of males on females is significantly steeper, providing evidence of greater evolutionary divergence in male size than in female size. Fitch (1978) noted that the high variation of SSD in *Sceloporus* lizards*,* and the implications of sexual and natural selection in order to explain the differences in body size between females and males. For lizard species in which body size often determines male mating success, males are typically larger than females (Cox et al., 2007). Body size often determines success in agonistic encounters, and it is correlated with dominance and territoriality (Carpenter, 1995; Martins, 1994; McMann, 1993; Molina‐Borja et al., 1998; Perry et al., 2004). However, in other species, females are larger than males, suggesting that fecundity selection may have favored the evolution of larger‐than‐average female body size (Cox et al., 2003; Zamudio, 1998). Furthermore, as *Sceloporus* lizards follow Rensch's rule, it can be argued that this allometric trend is the result of sexual selection favoring large male body size and that the relative impact of sexual selection on males has been stronger than fecundity selection on female body size (Fairbairn, 1997; Fairbairn et al., 2007; Pincheira‐Donoso & Tregenza, 2011).

The reconstruction of the evolution of SSD in *Sceloporus* lizards suggests that the ancestor and most of the extant species show a pattern of male‐biased SSD. This could indicate that directional sexual selection acting on males has been greater than the selection acting on female fecundity. Territoriality and aggressive behaviors are common in *Sceloporus*: These are mainly associated with defense of mates in males (Martins, 1994), and resources (e.g., food, water, perches) in both sexes (Cooper & Wilson, 2007; Martins, 1994; Vinegar, 1975c; Woodley & Moore, 1999). In general, larger individuals have an advantage when defending territories in agonistic encounters (Martins, 1994; Swierk, 2014). However, female‐biased SSD has evolved independently at least six times (Figure 3). Perhaps in these taxa, selection on fecundity has been stronger than sexual selection. Nonetheless, it is possible that in these species, sexual selection has also favored small male body size (see Cox et al., 2007; Olsson et al., 2002), albeit there is no clear pattern as to the ecological factors associated with the evolution of female‐biased SSD. These species, like other *Sceloporus* species that show male‐biased SSD, live in different environments, including tropical deciduous forest, grasslands, scrubland, woodlands, and open coniferous forests, and can be found from sea level up to elevations >4,000 m. Moreover, species showing female‐biased SSD are oviparous and viviparous (e.g., *undulatus* group versus *S. bicanthalis,* respectively), and with single or multiples clutches per reproductive season (e.g., *S. melanorhinus* versus *S. consobrinus,* respectively). The diversity of ecological and social factors provides opportunities for changes in the direction and magnitude of natural and sexual selection between and within species. However, the information available for female preference and agonistic interactions between males are, in the majority of species, severely scarce or absent (see Martins, 1994; Swierk, 2014).

Previous studies in Phrynosomatidae do not support Rensch′s rule (Cox et al., 2007). However, these results could be obscured by the large diversity in morphology, behavior, ecology, and life‐history traits between different lizards genera (Cox et al., 2003). In addition, these studies do not consider the phylogenetic relationship between the species (see Cox et al., 2007). Conversely, our results are similar to previous studies in the genus *Liolaemus* (Liolaemidae). The clutch/litter size increases as a function of female body size. Nonetheless, fecundity is not correlated with SSD, but *Lioalemus* species appear to follow Rensch's rule (Pincheira‐Donoso & Tregenza, 2011). Both *Sceloporus* and *Liolaemus* species occupy a great diversity of environments, along wide latitudinal and altitudinal ranges and showing great variation in morphological, ecological, behavior, and life‐history traits (Pincheira‐Donoso, Scolaro, & Sura, 2008; Sites et al., 1992). The similarity between our results and those reported in *Liolaemus* suggests that fecundity selection may have driven the divergence in female body size but that the diversifying effects of sexual selection may often exceed fecundity selection on females in both genera.

The genus *Sceloporus* includes more than 90 species and has been proposed as a group with an accelerated diversification rate (Bell, Smith, & Chiszar, 2003; Leaché, 2010; Wiens et al., 2010). *Sceloporus* lizards have colonized diverse niches throughout its distribution range, from northern Panama to southwestern Canada, and show one of the widest altitudinal ranges for reptiles. Due to the broad spread of niches, it is likely that the relative impact of natural and sexual selection has changed along novel environmental conditions, generating divergence from the optimum body size of females and males. In any case, the causal mechanisms associated with changes in the direction of SSD bias toward females in this group remain an open question that demand further investigation.

## Conflict of Interest

None declared.
