# Supplementary material for: The interplay between natural and sexual selection in the evolution of sexual size dimorphism in Sceloporus lizards (Squamata: Phrynosomatidae)
Source: Ecol Evol. 2017 Jan 13;7(3):905–17. doi: 10.1002/ece3.2572 (PMC5288261; doi:10.1002/ece3.2572)

**Appendix S1.**

The Table S1 and S2 showed the **G**enBank accession numbers for the 61 species used in the phylogenetic analyses. Nuclear and mitochondrial genes are listed alphabetically.

*Table S1. GenBank accession numbers of the eight nuclear genes used for the present study*

|  | BDNF | ECEL | PNN | PRLR | PTPN | R35 | RAG1 | TRAF6 |
| --- | --- | --- | --- | --- | --- | --- | --- | --- |
| *P_thalassinus* |  | GQ895767.1 | KP820518.1 | GQ896079.1 | GQ896211.1 | KP820536.1 | GQ896006.1 | GQ895940.1 |
| *S_adleri* |  |  |  |  |  |  |  |  |
| *S_aeneus* |  |  |  |  |  | KF437157.1 |  |  |
| *S_angustus* |  |  |  | GQ896082.1 | GQ896214.1 |  |  | GQ895944.1 |
| *S_arenicolus* | GQ464421.1 |  | KC853996.1 |  |  | GQ464645.1 | GQ464701.1 |  |
| *S_bicanthalis* | GQ464435.1 | KR360374.1 | GQ464603.1 | KR359866.1 | KR359920.1 | GQ464659.1 | GQ464715.1 | KR360099.1 |
| *S_chrysostictus* |  |  |  |  |  |  |  |  |
| *S_clarkii* | GQ464412.1 | GQ895769.1 | GQ464580.1 | GQ896083.1 | GQ896215.1 | GQ464636.1 | GQ464692.1 | GQ895945.1 |
| *S_couchi* | GQ464461.1 | GQ895770.1 | GQ464629.1 | GQ896084.1 | GQ896216.1 | GQ464685.1 | GQ464741.1 | GQ895946.1 |
| *S_cozumelae* |  |  |  |  |  |  |  |  |
| *S_cryptus* | GQ464415.1 |  | GQ464583.1 |  |  | GQ464639.1 | GQ464695.1 |  |
| *S_cyanogenys* |  |  |  |  |  |  |  |  |
| *S_dugesii* | GQ464439.1 |  | GQ464607.1 |  |  | GQ464663.1 | GQ464719.1 |  |
| *S_edwardtaylori* | GQ464414.1 |  | GQ464582.1 |  |  | GQ464638.1 | GQ464694.1 |  |
| *S_f_formosus* |  | GQ895771.1 |  | GQ896085.1 | GQ896217.1 |  |  | GQ895947.1 |
| *S_f_scitulus* | GQ464416.1 |  | GQ464584.1 |  |  | GQ464640.1 | GQ464696.1 |  |
| *S_gadoviae* | GQ464453.1 | GQ895772.1 | GQ464621.1 | GQ896086.1 | GQ896218.1 | GQ464677.1 | GQ464733.1 | GQ895948.1 |
| *S_graciosus* | GQ464422.1 | GQ895773.1 | GQ464590.1 | GQ896087.1 | GQ896219.1 | GQ464646.1 | GQ464702.1 | GQ895949.1 |
| *S_grammicus* | GQ464424.1 | GQ895774.1 | GQ464592.1 | GQ896088.1 | GQ896220.1 | GQ464648.1 | GQ464704.1 | GQ895950.1 |
| *S_grandaevus* | GQ464452.1 |  | GQ464620.1 |  |  | GQ464676.1 | GQ464732.1 |  |
| *S_horridus* | GQ464437.1 |  | GQ464605.1 |  |  | GQ464661.1 | GQ464717.1 |  |
| *S_hunsakeri* | GQ464427.1 |  | GQ464595.1 |  |  | GQ464651.1 | GQ464707.1 |  |
| *S_jarrovii* | GQ464440.1 |  | GQ464608.1 |  |  | GQ464664.1 | GQ464720.1 |  |
| *S_jalapae* | GQ464456.1 | GQ895775.1 | GQ464624.1 | GQ896089.1 | GQ896221.1 | GQ464680.1 | GQ464736.1 | GQ895951.1 |
| *S_licki* | GQ464428.1 |  | GQ464596.1 |  |  | GQ464652.1 | GQ464708.1 |  |
| *S_melanorhinus* | GQ464413.1 |  | GQ464581.1 |  |  | GQ464637.1 | GQ464693.1 |  |
| *S_mucronatus* | GQ464443.1 |  | GQ464611.1 |  |  | GQ464667.1 | GQ464723.1 |  |
| *S_omiltemanus* |  |  |  |  |  |  |  |  |
| *S_macdougalli* | GQ464442.1 |  | GQ464610.1 |  |  | GQ464666.1 | GQ464722.1 |  |
| *S_magister* | GQ464429.1 | GQ895777.1 | GQ464597.1 | GQ896091.1 |  | GQ464653.1 | GQ464709.1 | GQ895953.1 |
| *S_malachiticus* | GQ464417.1 |  | GQ464585.1 |  |  | GQ464641.1 | GQ464697.1 |  |
| *S_megalepidurus* | GQ464432.1 | GQ895778.1 | GQ464600.1 | GQ896092.1 | GQ896222.1 | GQ464656.1 | GQ464712.1 | GQ895954.1 |
| *S_merriami* | GQ464459.1 | GQ895779.1 | GQ464627.1 | GQ896093.1 | GQ896223.1 | GQ464683.1 | GQ464739.1 | GQ895955.1 |
| *S_minor* |  |  |  |  |  |  |  |  |
| *S_nelsoni* |  |  |  |  |  |  |  |  |
| *S_occidentalis* | GQ464448.1 |  | GQ464616.1 | KP820502.1 |  | GQ464672.1 | GQ464728.1 |  |
| *S_ochoterenae* | GQ464457.1 |  | GQ464625.1 |  |  | GQ464681.1 | GQ464737.1 |  |
| *S_olivaceus* | GQ464434.1 | GQ895780.1 | GQ464602.1 | GQ896094.1 | GQ896224.1 | GQ464658.1 | GQ464714.1 | GQ895956.1 |
| *S_orcutti* | GQ464430.1 |  | GQ464598.1 |  |  | GQ464654.1 | GQ464710.1 |  |
| *S_parvus* | GQ464462.1 | GQ895781.1 | GQ464630.1 | GQ896095.1 | GQ896225.1 | GQ464686.1 | GQ464742.1 | GQ895957.1 |
| *S_pictus* |  |  | GQ464601.1 |  |  | GQ464657.1 | GQ464713.1 |  |
| *S_poinsettii* | GQ464445.1 | GQ895782.1 | GQ464613.1 | GQ896096.1 | GQ896226.1 | GQ464669.1 | GQ464725.1 | GQ895958.1 |
| *S_pyrocephalus* | GQ464454.1 | GQ895783.1 | GQ464622.1 | GQ896097.1 | GQ896227.1 | GQ464678.1 | GQ464734.1 | GQ895959.1 |
| *S_s_caeruleo.* |  | GQ895785.1 |  |  | GQ896230.1 |  |  | GQ895962.1 |
| *S_s_spinosus* | GQ464438.1 |  | GQ464606.1 |  |  | GQ464662.1 | GQ464718.1 |  |
| *S_scalaris* | GQ464436.1 | GQ895784.1 | GQ464604.1 | GQ896098.1 | GQ896228.1 | GQ464660.1 | GQ464716.1 | GQ895960.1 |
| *S_siniferus* | GQ464460.1 |  | GQ464628.1 | GQ896099.1 | GQ896229.1 | GQ464684.1 | GQ464740.1 | GQ895961.1 |
| *S_smaragdinus* | EU085927.1 |  |  |  |  |  | EU085721.1 |  |
| *S_subpictus* | GQ464419.1 |  | GQ464587.1 |  |  | GQ464643.1 | GQ464699.1 |  |
| *S_torquatus* | GQ464446.1 | GQ895786.1 | GQ464614.1 | GQ896100.1 | GQ896231.1 | GQ464670.1 | GQ464726.1 | GQ895963.1 |
| *S_consobrinus* | GQ494864.1 |  | GQ494849.1 |  |  | GQ494834.1 | GQ494819.1 |  |
| *S_tristichus* | GQ494860.1 |  | GQ494845.1 |  |  | GQ494830.1 | GQ494815.1 |  |
| *S_undulatus* | GQ464449.1 | GQ895787.1 | GQ464617.1 | GQ896101.1 | GQ896232.1 | GQ464673.1 | GQ464729.1 | GQ895964.1 |
| *S_utiformis* | GQ464455.1 |  | GQ464623.1 |  | GQ896233.1 | GQ464679.1 | GQ464735.1 | GQ895965.1 |
| *S_variabilis* | GQ464464.1 | GQ895788.1 | GQ464632.1 | GQ896102.1 | GQ896234.1 | GQ464688.1 | GQ464744.1 | GQ895966.1 |
| *S_virgatus* | GQ464450.1 |  | GQ464618.1 |  |  | GQ464674.1 | GQ464730.1 |  |
| *S_woodi* | GQ464451.1 |  | GQ464619.1 |  |  | GQ464675.1 | GQ464731.1 |  |
| *U_bicarinatus* | JN648384.1 | GQ895790.1 |  | GQ896104.1 | GQ896236.1 |  | GQ896030.1 | GQ895968.1 |
| *U_graciosus* | JN648393.1 | GQ895791.1 |  | GQ896105.1 |  |  | JN648493.1 | GQ895969.1 |
| *U_nigricaudus* | GQ464465.1 | GQ895792.1 | GQ464633.1 | GQ896106.1 | GQ896237.1 | GQ464689.1 | GQ464745.1 | GQ895970.1 |
| *U_ornatus* | KP820848.1 | GQ895793.1 | KP820515.1 | GQ896107.1 | GQ896238.1 | KP820533.1 | GQ896033.1 | GQ895971.1 |

*Table S2. GenBank accession numbers of the five mitochondrial genes used for the present study*

|  | 12S | 16S | ND1 | ND2 | ND4 |
| --- | --- | --- | --- | --- | --- |
| *P_thalassinus* | L40445.1 | L41451.1 | KP899454.1 | KP899454.1 | KP899454.1 |
| *S_adleri* | AF000799.1 | AF000839.1 | AY297519.1 | AY297519.1 | GQ895850.1 |
| *S_aeneus* |  |  |  |  | JN985666.1 |
| *S_angustus* | L40450.1 | L41457.1 | AF049859.1 | AF049859.1 | AF210360.1 |
| *S_arenicolus* | GQ464524.1 | AF000863.1 | GQ464468.1 |  | GQ464748.1 |
| *S_bicanthalis* | GQ464525.1 | AF000840.1 | GQ464469.1 |  | GQ464749.1 |
| *S_chrysostictus* | L40451.1 | L41458.1 |  |  | AF210367.1 |
| *S_clarkii* | GQ464527.1 | L41459.1 | GQ464471.1 | AY297511.1 | GQ464751.1 |
| *S_couchi* | GQ464528.1 | AF000829.1 | GQ464472.1 |  | GQ464752.1 |
| *S_cozumelae* | AF000790.1 | AF000830.1 |  |  |  |
| *S_cryptus* | GQ464529.1 | AF000842.1 | GQ464473.1 |  | GQ464753.1 |
| *S_cyanogenys* | DQ525893.1 | L41460.1 | AY297524.1 | AY297524.1 | DQ525868.1 |
| *S_dugesii* | GQ464530.1 | L41461.1 | GQ464474.1 |  | GQ464754.1 |
| *S_edwardtaylori* | GQ464531.1 |  | GQ464475.1 |  | GQ464755.1 |
| *S_f_formosus* | L40455.1 | L41462.1 | AY297498.1 | AY297498.1 |  |
| *S_f_scitulus* | GQ464532.1 |  | GQ464476.1 |  | GQ464756.1 |
| *S_gadoviae* | GQ464533.1 | AF000836.1 | GQ464477.1 |  | GQ464757.1 |
| *S_graciosus* | GQ464534.1 | L41463.1 | GQ464478.1 | AF049860.1 | GQ464758.1 |
| *S_grammicus* | GQ464535.1 | L41464.1 | GQ464479.1 | AY297509.1 | GQ464759.1 |
| *S_grandaevus* | GQ464536.1 |  | GQ464480.1 |  | GQ464760.1 |
| *S_horridus* | GQ464538.1 | AF000844.1 | GQ464482.1 |  | GQ464762.1 |
| *S_hunsakeri* | GQ464539.1 | AF000845.1 | GQ464483.1 | AY297506.1 | GQ464763.1 |
| *S_jarrovii* | GQ464541.1 | L41465.1 | GQ464485.1 | AY297512.1 | GQ464765.1 |
| *S_jalapae* | GQ464540.1 | AF000837.1 | GQ464484.1 | AY297504.1 | GQ464764.1 |
| *S_licki* | GQ464542.1 | AF000848.1 | GQ464486.1 |  | GQ464766.1 |
| *S_melanorhinus* | GQ464549.1 | AF000852.1 | GQ464493.1 |  | GQ464773.1 |
| *S_mucronatus* | GQ464551.1 | DQ525902.1 | GQ464495.1 | AY297497.1 | GQ464775.1 |
| *S_omiltemanus* |  | AF000888.1 |  |  | AF154233.1 |
| *S_macdougalli* | GQ464544.1 | AF000849.1 | GQ464488.1 |  | GQ464768.1 |
| *S_magister* | GQ464546.1 | L41466.1 | GQ464490.1 | AF528741.1 | GQ464770.1 |
| *S_malachiticus* | GQ464547.1 | L41467.1 | GQ464491.1 | AY297518.1 | GQ464771.1 |
| *S_megalepidurus* | GQ464548.1 | AF000862.1 | GQ464492.1 |  | GQ464772.1 |
| *S_merriami* | GQ464550.1 | L41468.1 | GQ464494.1 | AY297520.1 | GQ464774.1 |
| *S_minor* | DQ525891.1 | AF000866.1 |  |  | DQ525872.1 |
| *S_nelsoni* |  |  |  |  | AF210351.1 |
| *S_occidentalis* | GQ464552.1 | AB079242.1 | GQ464496.1 | AY297515.1 | GQ464776.1 |
| *S_ochoterenae* | GQ464553.1 | AF000853.1 | GQ464497.1 | AF528743.1 | GQ464777.1 |
| *S_olivaceus* | GQ464554.1 | L41471.1 | GQ464498.1 | AY297521.1 | GQ464778.1 |
| *S_orcutti* | GQ464555.1 | L41472.1 | GQ464499.1 | AY297508.1 | GQ464779.1 |
| *S_parvus* | GQ464558.1 | AF000832.1 | GQ464502.1 |  | GQ464782.1 |
| *S_pictus* | GQ464559.1 | AF000831.1 | GQ464503.1 | AY297500.1 | GQ464783.1 |
| *S_poinsettii* | GQ464560.1 | L41473.1 | GQ464504.1 | AY297510.1 | GQ464784.1 |
| *S_pyrocephalus* | GQ464561.1 | AF000833.1 | GQ464505.1 | AY297502.1 | GQ464785.1 |
| *S_s_caeruleo.* | EF025755.1 | AF000864.1 |  |  | EF025748.1 |
| *S_s_spinosus* | EF025756.1 | L41475.1 | GQ464509.1 | AY297525.1 | EF025749.1 |
| *S_scalaris* | GQ464562.1 | L41474.1 | GQ464506.1 | AF528742.1 | GQ464786.1 |
| *S_siniferus* | GQ464563.1 | AF000834.1 | GQ464507.1 | AY297494.1 | GQ464787.1 |
| *S_smaragdinus* | EU086043.1 | AF000855.1 | AY297517.1 | AY297517.1 | EU085838.1 |
| *S_subpictus* | GQ464565.1 | AF000857.1 | GQ464511.1 |  | GQ464791.1 |
| *S_torquatus* | DQ525888.1 | DQ525905.1 | GQ464513.1 |  | GQ464793.1 |
| *S_consobrinus* | AF000820.1 | AF000860.1 | AF440079.1 |  |  |
| *S_tristichus* | AF440068.1 | AF440024.1 | EF031910.1 |  |  |
| *S_undulatus* | GQ464570.1 | AF000886.1 | GQ464514.1 | AY297514.1 | GQ464794.1 |
| *S_utiformis* | GQ464571.1 | HM012692.1 | GQ464515.1 | AF528740.1 | GQ464795.1 |
| *S_variabilis* | GQ464573.1 | L41479.1 | GQ464517.1 | AY297507.1 | GQ464797.1 |
| *S_virgatus* | GQ464574.1 | L41480.1 | GQ464518.1 | AY297516.1 | GQ464798.1 |
| *S_woodi* | GQ464575.1 | AF000858.1 | GQ464519.1 | AY297513.1 | GQ464799.1 |
| *U_bicarinatus* |  | HM012694.1 | JN648463.1 | JN648424.1 | AF210338.1 |
| *U_graciosus* | L41433.1 | L41484.1 | AF049862.1 | JN648433.1 | GQ895841.1 |
| *U_nigricaudus* | GQ464577.1 | KP091282.1 | GQ464521.1 | JN648426.1 | GQ464801.1 |
| *U_ornatus* | AF194247.1 | L41487.1 | JN648470.1 | AY297493.1 | AY141065.1 |

###### Phylogeny Estimation and divergence times

The phylogeny was estimated based on a concatenate data matrix of 11,113 characters. The best nucleotide substitution models for the nuclear genes are showed in Table S3.

*Table S3. Nucleotide substitutions models selected for eight nuclear genes*

| Nuclear gene data partitions | Nucleotide substitution model |
| --- | --- |
| **BDNF** | K80 +I |
| **ECEL** | K80 +Γ |
| **PNN** | GTR + Γ |
| **PRLR** | K80 + Γ |
| **PTPN** | HKY + Γ |
| **R35** | K80 + I + Γ |
| **RAG1** | HKY + Γ |
| **TRAF6** | K80 + Γ |

For the mitochondrial genes, the best scheme and nucleotide substitution models are showed in Table S4.

*Table S4. Nucleotide substitutions models selected for five mitochondrial genes*

| Mitochondrial gene data partitions | Nucleotide substitution model |
| --- | --- |
| **12S** | GTR + I + Γ |
| **16S** | GTR + Γ |
| **ND1** |  |
| *1st position* | GTR + I + Γ |
| *2nd position* | HKY + I + Γ |
| **ND2** |  |
| *1st position* | GTR + I + Γ |
| *2nd position* | HKY + I + Γ |
| **ND4** |  |
| *1st position* | GTR + I + Γ |
| *2nd position* | GTR + Γ |

For converted the molecular branch lengths from the Bayesian analysis to units of time we used a penalized likelihood method with the chronopl() function in R package ‘ape’ (Sanderson 2002). The penalized likelihood is a semiparametric approach that allows differential rates of evolution across the phylogenetic tree. The method used a trade-off between a parametric formulation where each branch has its own rate, and a nonparametric term where changes in rates are minimized between contiguous branches. A smoothing parameter (lambda) controls this trade-off. If lambda = 0, then the parametric component dominates and rates vary as much as possible among branches, whereas for increasing values of lambda, the variation are smoother to tend to a clock-like model (same rate for all branches). We used a lambda value of 0.5 for our analysis which maintained an intermediated value in the substitution rates.

Figure 1S. Phylogeny of 56 *Sceloporus* species and five outgroup taxa based on a combined, and partitioned analysis of eight nuclear genes and five mitochondrial genes. The maximum likelihood values are show in the nodes.


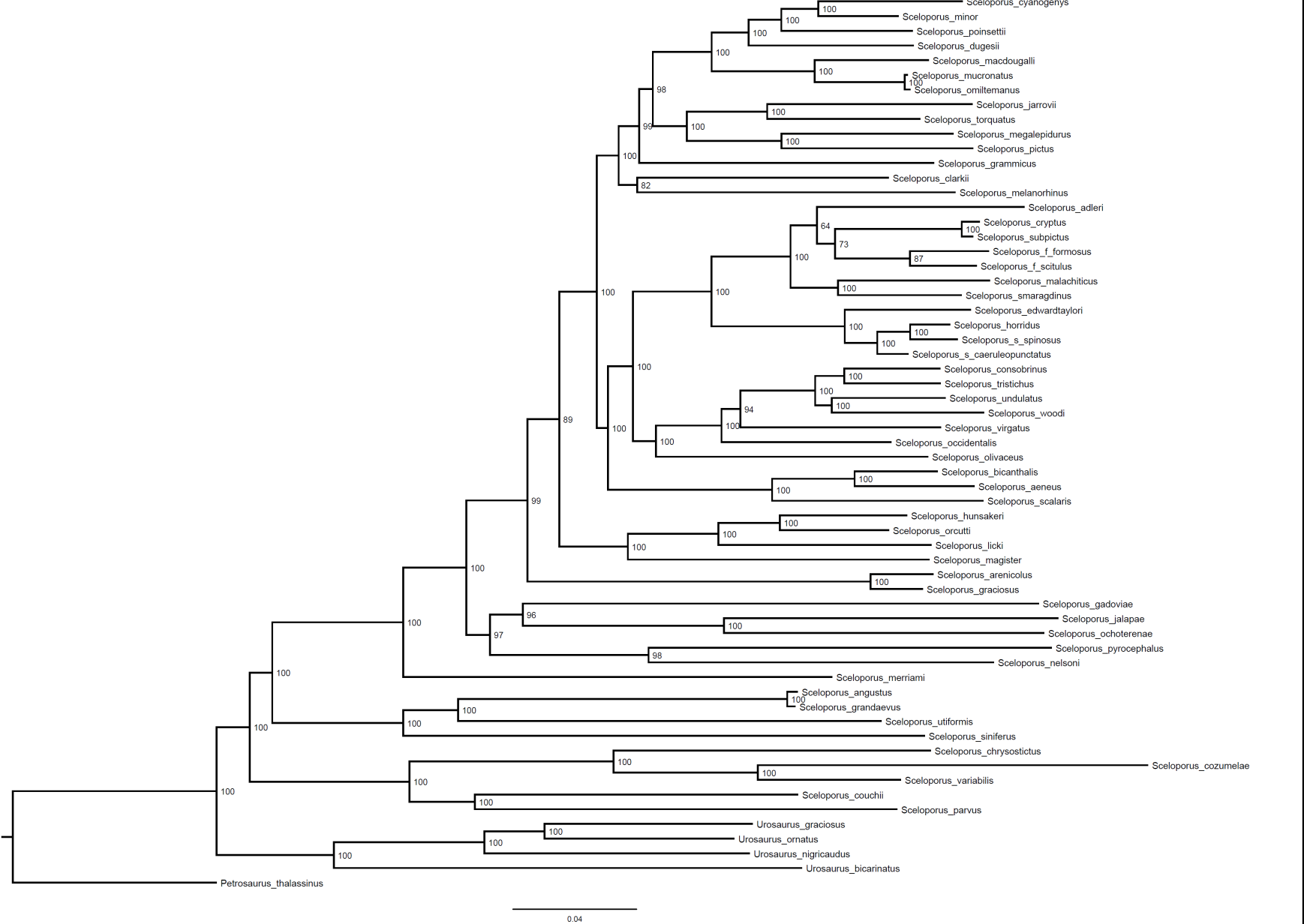

Supplement: Supplementary file 1 [file ECE3-7-905-s001.docx]
